# Correlation of the lung microbiota with metabolic profiles in bronchoalveolar lavage fluid in HIV infection

**DOI:** 10.1186/s40168-016-0147-4

**Published:** 2016-01-20

**Authors:** Sushma K. Cribbs, Karan Uppal, Shuzhao Li, Dean P. Jones, Laurence Huang, Laura Tipton, Adam Fitch, Ruth M. Greenblatt, Lawrence Kingsley, David M. Guidot, Elodie Ghedin, Alison Morris

**Affiliations:** Pulmonary Medicine, Department of Veterans Affairs Medical Center, 1670 Clairmont Rd, Mailstop 151p, Decatur, 30033 GA USA; Department of Medicine, Division of Pulmonary, Allergy and Critical Care, Emory University, Atlanta, GA USA; Department of Medicine, HIV/AIDS Division and Division of Pulmonary and Critical Care Medicine, University of California, San Francisco, Medicine, San Francisco, CA USA; Department of Computational and Systems Biology, University of Pittsburgh School of Medicine, Pittsburgh, PA USA; Department of Biology, Center for Genomics and Systems Biology, and Global Institute of Public Health, New York University, New York, NY USA; Department of Medicine, Division of Pulmonary, Allergy, and Critical Care Medicine, University of Pittsburgh School of Medicine, Pittsburgh, PA USA; Department of Clinical Pharmacy, University of California, San Francisco, Medicine, San Francisco, CA USA; Departments of Infectious Diseases and Microbiology and Epidemiology, GSPH, University of Pittsburgh, Pittsburgh, PA USA

**Keywords:** HIV, Lung, Metabolomics, Microbiota, Bronchoalveolar lavage

## Abstract

**Background:**

While 16S ribosomal RNA (rRNA) sequencing has been used to characterize the lung’s bacterial microbiota in human immunodeficiency virus (HIV)-infected individuals, taxonomic studies provide limited information on bacterial function and impact on the host. Metabolic profiles can provide functional information on host-microbe interactions in the lungs. We investigated the relationship between the respiratory microbiota and metabolic profiles in the bronchoalveolar lavage fluid of HIV-infected and HIV-uninfected outpatients.

**Results:**

Targeted sequencing of the 16S rRNA gene was used to analyze the bacterial community structure and liquid chromatography-high-resolution mass spectrometry was used to detect features in bronchoalveolar lavage fluid. Global integration of all metabolic features with microbial species was done using sparse partial least squares regression. Thirty-nine HIV-infected subjects and 20 HIV-uninfected controls without acute respiratory symptoms were enrolled. Twelve mass-to-charge ratio (*m/z)* features from C18 analysis were significantly different between HIV-infected individuals and controls (false discovery rate (FDR) = 0.2); another 79 features were identified by network analysis. Further metabolite analysis demonstrated that four features were significantly overrepresented in the bronchoalveolar lavage (BAL) fluid of HIV-infected individuals compared to HIV-uninfected, including cystine, two complex carbohydrates, and 3,5-dibromo-l-tyrosine. There were 231 *m/z* features significantly associated with peripheral blood CD4 cell counts identified using sparse partial least squares regression (sPLS) at a variable importance on projection (VIP) threshold of 2. Twenty-five percent of these 91 *m/z* features were associated with various microbial species. Bacteria from families *Caulobacteraceae*, *Staphylococcaceae*, *Nocardioidaceae*, and genus *Streptococcus* were associated with the greatest number of features. Glycerophospholipid and lineolate pathways correlated with these bacteria.

**Conclusions:**

In bronchoalveolar lavage fluid, specific metabolic profiles correlated with bacterial organisms known to play a role in the pathogenesis of pneumonia in HIV-infected individuals. These findings suggest that microbial communities and their interactions with the host may have functional metabolic impact in the lung.

**Electronic supplementary material:**

The online version of this article (doi:10.1186/s40168-016-0147-4) contains supplementary material, which is available to authorized users.

## Background

Combination anti-retroviral therapy (ART) has reduced the incidence of opportunistic infections in individuals infected with human immunodeficiency virus (HIV), but certain pneumonias and chronic lung diseases remain important clinical problems. Subtle immune deficits may alter the microbial composition in the lung, and these microbes or their products may impact the host.

Culture-independent techniques, such as 16S ribosomal RNA (rRNA) gene sequencing, have been instrumental in identifying bacterial species present in the lung microbiome [1]. Alterations in complex microbial communities have been identified in many diseases compared to healthy individuals [2]; however, most studies of the lung microbiome have primarily described bacterial communities. These taxonomic analyses of microbial communities provide useful information, but have limited insights into the functional impact of the bacterial microbiome. Metabolomics is the scientific study of small-molecule by-products of host or microbial processes that are part of an integrated biosystems approach; they provide a unique molecular fingerprint for an individual or tissue. The metabolome, which represents a collection of small molecule features found within an organism, is dynamic and can reflect various physiological pathways including bacterial metabolism and the host inflammatory response. New high-throughput technologies enable metabolome-wide association studies (MWAS) that can support integrated models for personalized medicine by correlating specific metabolites with a specific disease.

There are limited data available on the metabolome of HIV-infected individuals, but metabolomic analyses can help elucidate interactions between inflammation and cellular metabolism [3]. A few studies have characterized the profile of metabolites present in oral wash samples [4] and cerebrospinal fluid [5]. Most recently, we used liquid chromatography-high-resolution mass spectrometry (LC-FTMS) to perform metabolomic analyses of bronchoalveolar lavage (BAL) fluid of otherwise healthy HIV-infected and HIV-uninfected individuals [6]. We demonstrated that overall metabolic profiles in BAL fluid were different between these two groups and showed that pyochelin, a siderophore produced by *Pseudomonas aeruginosa*, was elevated in the lungs of HIV-infected individuals compared to HIV-uninfected individuals [6]. While this study did not investigate microbial communities in the lung, many of the significant metabolites identified were not associated with any known human metabolites and therefore likely produced by microbial species. Given the potential metabolic products from bacteria to stimulate host metabolism, investigation of the relationship of metabolomics and the microbiota has the potential to provide novel insights into pathogenic processes.

Thus, we investigated the respiratory microbiota and global metabolic profiles in the BAL of HIV-infected and HIV-uninfected individuals to determine if specific metabolites and bacteria genera were associated with HIV infection. This study is the first to evaluate the global functional metabolic interactions between the microbial species in the lung and the metabolome in HIV-infected individuals.

## Results

### Demographics of HIV-infected and HIV-uninfected participants

Fifty-nine participants were enrolled (39 HIV-infected participants and 20 HIV-uninfected); 47 were used for the training set (32 HIV-infected and15 HIV-uninfected subjects) while 12 were used for the validation (test) set (7 HIV-infected and 5 HIV-uninfected). There were no differences in race, sex, or smoking status between the groups (Table 1). The majority of HIV-infected subjects (87.2 %) reported receiving ART at the time of study, and their median CD4 count was 600 cells/μl (interquartile range (IQR) 404–853). There was only one HIV-infected individual with a CD4 count below 200 cells/μl Additional file 1.

**Table 1:** Demographics of HIV-infected and HIV-uninfected participants

Variables	HIV (+), *n* = 39	HIV (−), *n* = 20	*p* value
Gender, *n* (% male)	31 (79.5)	15 (75.0)	0.69
Race, *n* (%)			0.51
White	20 (51.3)	13 (65)	
Black	18 (46.1)	7 (35)	
Other	1 (2.6)		
Ever smokers, *n* (%)	24 (61.5)	10 (50.0)	0.40
Current smokers, *n* (%)	10 (25.6)	5 (25.0)	0.82
CD4 cell count, ^a^median cells/μl (IQR)	600 (404–853)	1137 (767–1272)	0.008
CD4 count ≤200, *n* (%)^a^	1 (2.6)		
Currently taking antiretroviral therapy, *n* (%)	34 (87.2)		
Simpson’s Diversity Index, mean (SD)	0.71 (0.26)	0.81 (0.12)	0.96

^a^Sample size is different than above: HIV (+), *n* = 38 and HIV (−), *n* = 6

### Lung microbiota did not distinguish HIV-infected from HIV-uninfected individuals

**Fig. 1:**
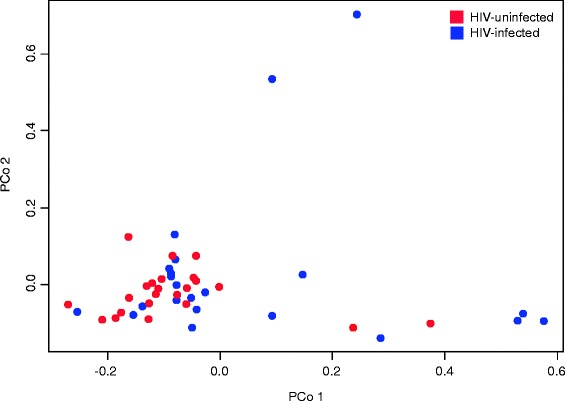
Principal coordinate analysis (PCoA) plot showing the clustering trend of bronchoalveolar (BAL) samples based on the weighted UniFrac distance. PCoA plot showing the clustering trend of bronchoalveolar (BAL) samples based on the weighted UniFrac distance between all OTUs identified in HIV-infected (*n* = 39) and HIV-uninfected (*n* = 20) individuals. In this cohort, the lung microbiota does not distinguish HIV status, but comparison of intra-group beta diversity showed a significant difference in UniFrac distances between the HIV-infected and HIV-uninfected subjects, suggesting a higher degree of heterogeneity among the microbial composition of the lower airways in HIV-infected subjects

Taxonomic assignment of the operational taxonomic units (OTUs) revealed a total of 153 bacterial taxa across all samples. The microbial lung communities in these participants fell into one of two “pulmotypes,” dominated by either the order *Actinomycetales* or the order *Bacteroidales*; in the latter, we see a combination of the family *Prevotellaceae* and the family *Streptococcaceae.* These types did not sort by HIV status. When using principal coordinate analysis (PCoA) of the weighted UniFrac distance between all OTUs identified, the samples also did not cluster or separate by HIV status (Fig. 1). Unlike principal component analysis (PCA), PCoA can use the weighted UniFrac distance between samples, which incorporates both phylogenetic and compositional differences in communities. Comparison of intra-group beta diversity showed a significant difference in UniFrac distances between the HIV-infected and HIV-uninfected subjects, suggesting a higher degree of heterogeneity among the microbial composition of the lower airways in HIV-infected subjects. Furthermore, there was no difference in biodiversity, as measured by Simpson’s Diversity Index (Table 1). There was also no difference in community composition based on institution where the samples were collected. These findings indicate that there were no significant differences between the lung microbiota of our HIV-infected and HIV-uninfected groups.

### Metabolomics of bronchoalveolar lavage fluid distinguish HIV-infected from HIV-uninfected individuals

**Fig. 2:**
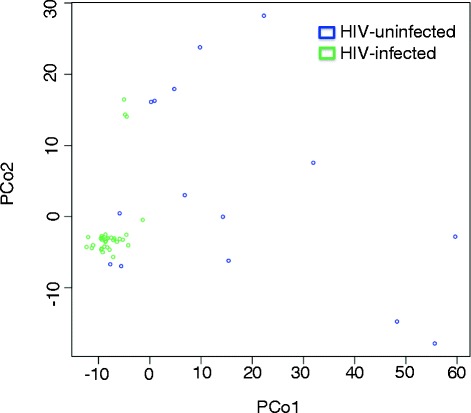
Principal coordinate analysis (PCoA) analysis of 12 *m/z* features from C18 chromatography. PCoA analysis of 12 *m/z* features from C18 chromatography shows that the lung metabolome does distinguish HIV-status and there is decreased variability in the metabolome in HIV-infected subjects compared to HIV-uninfected subjects

We compared the metabolomics features found in the BALs of the HIV-infected and HIV-uninfected groups. Of the 5930 mass-to-charge ratio (*m/z*) features identified by C18 chromatography, 12 were significantly different between the HIV-infected and uninfected individuals at a false discovery rate (FDR) = 0.2 (Appendix 1). Unadjusted raw data for these 12 features were also evaluated to determine statistical significance. A logistic regression model using the top three features (*p* < 0.0002; FDR 0.05) as predictors gave an area under the curve (AUC) of 0.89 and 0.80, respectively, for the 47 training samples and 12 independent test samples (7 HIV-infected vs. 5 HIV-uninfected) used only for validation (see Additional file 2). The 10-fold cross-validation classification accuracy for the 47 training samples was 90.8 %, and the overall classification accuracy for the 12 test samples was 83.3 % using the logistic regression model. Further, PCoA of 12 *m/z* features from C18 chromatography shows not only that the lung metabolome distinguishes HIV status but also that there is decreased variability in the metabolome among HIV-infected subjects compared to HIV-uninfected subjects (Fig. 2). Using the PCoA analysis allowed for a direct comparison to the microbiota data. Network analysis of the 12 differentiating *m/z* features based on Pearson correlation showed an additional 79 *m/z* features that correlated with these features with a |*r*| > 0.40 at FDR = 0.2.

Further metabolite analysis demonstrated that four features were significantly overrepresented in the BAL fluid of HIV-infected individuals compared to HIV-uninfected, including cystine, two complex carbohydrates and 3,5-dibromo-l-tyrosine (see Additional file 2). Cystine was measured in the BAL fluid using LC-FTMS, as done previously [3] and showed increased concentrations among HIV-infected subjects compared to HIV-uninfected subjects; this increase was however not statistically significant (7.3 (IQR 3.2–16.9) μM vs. 4.3 (IQR 3.6–9.7) μM, *p* = 0.53).

**Fig. 3:**
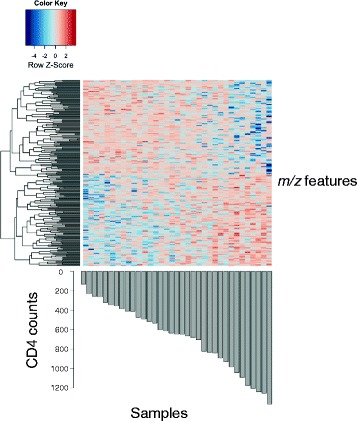
Association of peripheral CD4 cell counts with metabolomic features using sPLS at a variable importance on projection (VIP) threshold of 2. Association of peripheral CD4 cell counts with metabolomic features shows that 231 *m/z* features were found to be significantly associated with CD4 count using partial least squares (PLS) regression at variable importance on projection threshold of 2. Metabolite annotation and pathway enrichment analysis using *Mummichog* mapped these 231 *m/z* features to inflammatory pathways, including fatty acid activation and arginine metabolic pathways

There were 231 *m/z* features significantly associated with peripheral blood CD4 cell counts using sparse partial least squares regression (sPLS) at a variable importance on projection (VIP) threshold of 2 (Fig. 3). One-way hierarchical cluster analysis (HCA) showed that these features were grouped into 58 clusters based on the CD4 cell count. Metabolite annotation and pathway enrichment analysis using *Mummichog* mapped these 231 *m/z* features to a number of inflammatory pathways, including fatty acid activation (Table 2).

**Table 2:** Metabolite annotation and pathway enrichment analysis using Mummichog for 231 m/z features associated with CD4 cell count

Pathways	Pathway size	*p* value
Vitamin E metabolism	30	0.002
Urea cycle/amino group metabolism	37	0.004
Fatty acid activation	23	0.005
Arginine and proline metabolism	30	0.01
Aspartate and asparagine metabolism	55	0.02
Starch and sucrose metabolism	16	0.02
Linoleate metabolism	18	0.03
Fatty acid metabolism	22	0.04
Butanoate metabolism	23	0.04

### Integration of microbiome and metabolome

**Fig. 4:**
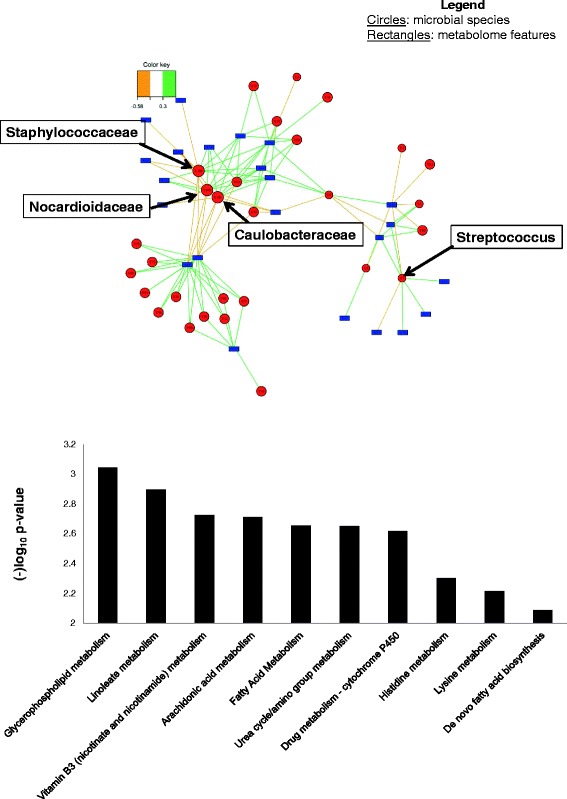
**a** Three-way relationship between metabolome, HIV, and microbiota integration of HIV-specific *m/z* features with microbiota data at correlation of 0.3. This figure shows that of the 91 *m/z* features associated with HIV, 23 were also found to be associated with 29 microbial genera at a correlation of 0.30; *red circles* microbial families and genus, *blue rectangles* metabolome features. **b** Pathway analysis using *m/z* features that were associated with *Staphylococcaceae* in the global network. Using *Mummichog*, metabolite annotation and pathway enrichment analysis was done using the *m/z* features that were associated with *Staphylococcaceae* (as the representative bacteria since pathways for *Caulobacteraceae* and *Nocardioidaceae* were very similar). The *y*-axis represents the enrichment significance of the metabolic pathways. This analysis shows that the pathways most affected included lineolate, glycerophospholipid, and fatty acid metabolism

Global integration using sPLS regression of all 5930 *m/z* features with the 153 microbial taxa identified showed that 109 microbial genera were associated with all features. Of the 91 *m/z* features associated with HIV, 23 were also found to be associated with 29 microbial genera at a correlation of 0.30 (Fig. 4a). The bacteria associated at the highest correlation with the greatest number of these 23 features included the families *Caulobacteraceae* (11 features), *Staphylococcaceae* (12 features), *Nocardioidaceae* (12 features), and the genus *Streptococcus* (7 features). *Streptococcus* and its associated *m/z* features were in a separate network compared to the other three bacterial families, as illustrated in the network analysis (Fig. 4b). Using *Mummichog*, metabolite annotation and pathway enrichment analysis was done using the *m/z* features that were associated with these three bacteria families—*Staphylococcaceae*, *Caulobacteraceae*, and *Nocardioidaceae. Staphylococcaceae* is shown in Fig. 4b as the representative family since pathways were similar for all three. This analysis shows that the metabolic pathways affected most significantly included lineolate, glycerophospholipid, and fatty acid metabolism. Pathway analysis could not be done for *Streptococcus* given the limited number of features.

## Discussion

We investigated the correlation between the lung microbiota and global metabolic pathways in the BAL of HIV-infected individuals to determine if specific features were altered in relation to HIV infection and to the microbial composition in the lung. The interactions of microorganisms with the host and the environment could lead to the development of a variety of diseases and contribute to their severity. Metabolomic analyses allow us to measure both metabolic changes in the host and in bacteria. A recent study in cystic fibrosis patients identified significant relationships among *P. aeruginosa*-isolate growth behavior, forced expiratory volume in one second, and differing metabolomics clusters [7], suggesting that bacterial species in the lungs are affecting metabolic composition and potentially resulting in adverse clinical outcomes. We previously found that metabolomic profiles in BAL fluid differentiated HIV-infected from HIV-uninfected individuals [6], but it was unclear if those patterns related to bacterial communities. The current study has combined these two modalities and identified a number of clinically relevant features that are altered with respect to specific lung microbial communities in HIV-infected individuals.

We found that specific metabolic pathways were not only associated with HIV status but specific metabolites were also increased in the BAL fluid of HIV-infected individuals compared to HIV-uninfected individuals, one of the most intriguing being cystine. Cysteine, an essential amino acid, is a rate-limiting component of glutathione synthesis and plays an important role in maintaining the detoxification of free radicals and reactive oxygen species [8, 9]. It is also an integral part of fatty acid synthesis [10]. Oxidative stress can be generated through several mechanisms including the oxidation of extracellular thiols, in particular cysteine to cystine [9], and HIV infection is known to cause oxidative stress [11–13]. Cysteine supply is limiting for important lymphocyte functions and impaired in HIV [14, 15]. In fact, treatment with N-acetylcysteine has been studied in ART-naïve HIV-infected patients [16, 17] and shown relative increases in CD4+ T cell numbers.

In our study, we observed that oxidized cysteine, or cystine, concentrations were increased in HIV-infected individuals and that CD4 counts were also associated with fatty acid metabolism. Further, fatty acid metabolism in addition to glycerophospholipid and linoleate metabolic pathways also correlated with the lung microbial species in these HIV-infected individuals; these have previously been shown to be altered systemically in HIV-infected individuals [18]. High dietary linoleic acid can lead to excessive prostaglandin-E2 production, which can depress cellular immunity specifically associated with the Th1 subset of T cells, which is also reduced in HIV infection [19, 20]. Alterations in surfactant glycerophospholipid have been seen in AIDS patients with acute *Pneumocystis* pneumonia [21]. Most recently, using combined metabolomic and qRT-PCR analyses, HIV-1 Tat protein was noted to cause significant alterations in glycerophospholipid metabolism [22]. These data suggest that HIV infection alters these metabolic pathways systemically. We now show that these perturbations, which are associated with pathogenic bacteria, are also present in the lung.

The bacterial organisms associated with the highest number of metabolic features—*Caulobacteraceae*, *Staphylococcaceae*, and *Nocardioidaceae—*are known causative organisms in the pathogenesis of pneumonia in HIV-infected patients. *Staphylococcaceae* are gram-positive organisms that include the genus *Staphylococcus*. Together with *Streptococcus,* these are two of the most frequent organisms seen in the era of ART and are often responsible for the greatest incidence of community-acquired pneumonia in HIV-infected adults and children [23]. Similarly, *Nocardioidaceae* is a family of bacteria with several genera including *Norcardia* and *Rhodococcus. Norcardia* are gram-positive organisms with partial acid-fast branching filaments that can be isolated in the soil worldwide, while *Rhodococcus equi* is a gram-positive coccobacillus that can also cause pneumonia in HIV-infected individuals [24]. *Caulobacteracea*e, an alpha-Proteobacteria, is common in aquatic and soil environments. It was recently shown that HIV-infected individuals who use cocaine display intestinal dysbiosis resulting in an abundance of *Proteobacteria* in their feces compared to HIV-uninfected controls [25]. It is possible that this organism, although mainly found in the gut, may be important for HIV pathogenesis in the lung as well. The *Caulobacteraceae* were not found in the reagent controls or in the relevant BAL controls.

**Fig. 5:**
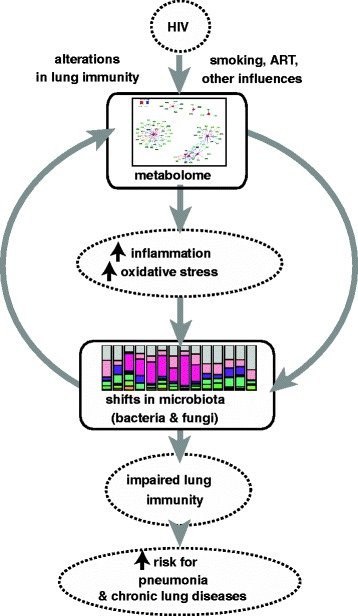
Overall schema. Overall schematic that postulates that HIV infection alters metabolomic profiles in lung (inflammatory and oxidant pathways) which causes a shift in the functional properties of the lung microbiome and/or in the host response to the lung microbiome which may lead to an increased risk of lung infection or to chronic lung disease associated with HIV

Based on our results, we postulate that HIV infection alters inflammatory and oxidant metabolic pathways within the lung, which then shift the functional properties of the lung microbiome and/or the host response to the lung microbiome (Fig. 5). The resulting increase in inflammation and oxidative stress may further impair lung host immunity leading to an increased risk of pneumonia or to chronic lung disease associated with HIV. In addition, alterations in redox balance may further influence the function of the microbiome and metabolome leading to a dynamic imbalance within the lung.

This study has several limitations. With respect to the microbiota data, we did not explicitly account for contamination (from the environment or upper airways). Although some investigators have noted that BAL fluid samples are not affected by upper airway contamination [26], others have not corroborated this finding [2]. However, by limiting our analyses to the 153 genera with >5 % relative abundance in all BAL samples, we were able to separate the BAL samples from the reagent controls. Another limitation is that this study only focuses on bacterial communities while other organisms, such as fungi, may also be important [27]. With respect to metabolomics, this study does not distinguish microbial from host metabolic products; however, given the prominent associations between the lung microbiota and metabolic profiles, it demonstrates potentially important relationships for future study. In addition, authenticated standards are not available for many of the features we identified, and so the 91 *m/z* features of interest have not all been validated. Metabolome data was also not adjusted for BAL or serum urea, which could have introduced error. Further, although 12 *m/z* features were significantly different between HIV-infected individuals and controls, these results were marginally significant at a FDR of 20 % and should be interpreted with some caution.

## Conclusions

In summary, we have performed the first study to investigate the respiratory microbiome-metabolome interaction in HIV-infected individuals. We found 12 metabolic features in BAL that differentiated between HIV-infected and HIV-uninfected participants, and another 79 features were identified in network analysis. Further metabolite analysis demonstrated that cystine was one of four features that were significantly overrepresented in the BAL fluid of HIV-infected individuals compared to HIV-uninfected and cystine concentrations were increased among HIV-infected subjects compared to HIV-uninfected subjects, albeit not statistically significant. Metabolome associations with CD4 counts also showed alterations in inflammatory pathways. Several HIV-differentiating metabolites, including those involved in inflammatory pathways, correlated with lung microbes known to cause lung infections. Further investigation into other aspects of innate lung immunity and the interaction between these organisms and the host immune response will be critical as this field moves forward. These findings may lead directly to a better understanding of the interactions of microbial communities, host physiology, and lung disease in HIV.

## Methods

### Study population

Fifty-nine participants from the Pittsburgh Lung HIV cohort were recruited to undergo bronchoscopy at the University of Pittsburgh, the University of California San Francisco, and the University of California Los Angeles [28, 29]. The lung HIV cohort enrolled a total of 396 HIV-infected and HIV-uninfected individuals from 2008 through 2011 from the Multicenter AIDS Cohort Study (MACS) and the Women’s Interagency HIV Study (WIHS) cohorts. Inclusion criteria for the bronchoscopy study included the absence of acute respiratory symptoms or fever in the previous four weeks and use of systemic antimicrobials (excluding antiretroviral treatments) or immunosuppressive medications within 6 months. We obtained clinical and demographic data by standardized subject interview and medical record review. Written informed consent was obtained from all participants after approval of human subjects’ protection protocols from review boards of the University of Pittsburgh, the University of California San Francisco, the University of California Los Angeles, and Emory University. The study was registered at clinicaltrials.gov (NCT00870857). Microbiota data from a subset of these individuals has been previously reported as part of the NHLBI’s Lung HIV Microbiome Project collaboration [30].

### Sample collection

Subjects were asked to fast and to refrain from smoking for at least 12 h before sample collection. Bronchoscopy with BAL was performed according to standardized procedures designed to minimize oral contamination [31]. BAL was performed by sequentially instilling and then withdrawing 50 ml aliquots of sterile normal saline up to a total of 200 ml into the right middle lobe or lingula.

### Sample processing and sequencing

DNA was extracted from samples using the Power Soil Extraction kit (Mo Bio, USA). Sequencing was performed using the Roche 454 FLX Titanium platform with primers for the amplification of hypervariable regions 1 through 3 (V1–3) of the 16S rRNA gene, as previously described [2]. Sequence reads were trimmed and analyzed using the QIIME software package [32]. These were then clustered into OTUs and used to characterize the diversity of the lung microbiota in the BAL. Specifically, to estimate the richness and evenness of the microbial community for each BAL sample, we calculated Simpson’s Diversity Index. To determine the phylogenetic and compositional differences between the microbial communities across the samples, we calculated weighted UNIFRAC distances [33] between pairs of samples. Intra-group beta diversities were also compared. Taxonomic assignment of the OTUs was performed down to the genus level, and genera with less than 5 % relative abundance in all samples were removed, leaving a total of 153 microbial genera across samples.

### Metabolomics

Samples were analyzed by LC-FTMS, as previously described [3]. *m/z* features were collected by a Thermo Q Exactive Hybrid Quadrupole-Orbitrap Mass Spectrometer (Thermo Fisher, San Diego, CA) from *m/z* 85 to 1275 over 10 min. Peak extraction, noise removal, and quantification of ion intensities were performed by an adaptive processing software package (apLCMS) with xMSanalyzer designed for use with LC-FTMS data [34]. For clarification, data extracted on *m/z* features, which are not definitively identified metabolites, will be referred to as “features” and not metabolites. See Additional file 2.

### Biostatistics and bioinformatics

The major predictor variable was HIV infection status. Two-sample Wilcoxon rank-sum tests were performed to compare continuous demographic characteristics between HIV-infected and HIV-uninfected individuals. Pearson chi-square tests were used to compare categorical characteristics. Statistical analyses were conducted in NCSS statistical software, except as indicated below. All reported *p* values were two-sided; and *p*-values of less than 0.05, corrected for multiple hypotheses testing as necessary, were considered statistically significant.

High-resolution mass spectral data were filtered to include only *m/z* features where at least one group (HIV-infected or HIV-uninfected) had values for 50 % of the samples. A total of 5930 *m/z* features were selected and used for all statistical analyses. LIMMA package [35] in R (linear models for microarray data) in Bioconductor was used to identify differentially expressed features at a significance threshold of 0.2 after Benjamini-Hochberg FDR adjustment. Two-way HCA was performed to identify clusters of individuals associated with discriminating clusters of features. Principal component analysis using Pirouette version 4.0 (InfoMetrix) was performed, as previously described [36]. Metabolite annotation and pathway enrichment analysis was done using *Mummichog* software implementation of an approach that uses the collective power of combining metabolite identification and metabolic pathway/network analysis into one site. The sPLS regression method [37] implemented in the R package mixOmics was used to perform integration and visualization of microbiome × metabolome associations. See Additional file 2.

### Availability of data

The sequence data supporting the results of this study is available in NCBI sequence read archive (SRA) under accession SRP065274.

## Appendix 1

**Table 3:** C18 Chromatography table of differentiating metabolites between HIV-infected and HIV-uninfected participants

Input mass	Adduct	Mass	Database match^a^	Formula	*p*_(Limma)_
241.0306778	[M + H]+	240.0238	Cystine	C6H12N2O4S2	0.001
1033.273196	[M + H-2H2O]+	1068.2864	2-O-beta-d-Glucopyranuronosyl-d-mannose	C36H60O36	0.03
1041.978368	[M + H]+	1040.9711	TG(21:0/22:1(13Z)/22:1(13Z))[iso3]	C68H128O6	0.0003
354.9312674	[M + NH4]+	336.8949	3,5-Dibromo-l-tyrosine	C9H9Br2NO3	0.0001
570.3873606	[M + Na]+	547.4002	PC(O-3:1(1E)/O-18:1(9Z))[S]	C29H58NO6P	0.0007
1189.600044	[M + H]+	1188.5928	Camelliasaponin A1	C58H92O25	0.0009
1203.845241	[M + H]+	1202.8380	GalNAcbeta1-4Galbeta1-4Glcbeta-Cer(d18:1/26:1(17Z))	C64H118N2O18	2.77*10^−5^
481.2527897	[M + Na]+	458.2603	N-(4-benzenesulfonamide) arachidonoyl amine	C26H38N2O3S	0.0026
541.8433866	No match				0.0025
393.2172688	[M + H]+	392.2100	Indacaterol	C24H28N2O3	0.0022
529.8652782	No match				9.96*10^−7^
272.1645049	[M + H]+	271.1572	Apoatropine	C17H21NO2	0.0023

^a^Matched in Metin at 10 ppm

## Additional files

Additional file 1:
**Bal public dataset.** (XLSX 12 kb) Additional file 2: Figures S1 and S2.
**Figure S1.** Receiver operating characteristic (ROC) curve using the top discriminatory features between HIV-infected (*n* = 32) and HIV-uninfected (*n* = 15) individuals. The top three features (*p* < 0.0002; FDR 0.05) were used as predictors in a logistic regression model that gave an area under the curve (AUC) = 0.89 and a 10-fold cross-validation classification accuracy of 90.8 % for the 47 training samples and AUC of 0.8 and classification accuracy of 83.3 % for the 12 test set samples. *Dotted line* training set (*n* = 47). *Solid line* validation (test) set (*n* = 12). Figure S2. Metabolomics of bronchoalveolar (BAL) fluid of HIV-infected individuals compared to HIV-uninfected. Further metabolite analysis demonstrates that four features were significantly over-represented in the bronchoalveolar lavage (BAL) fluid of HIV-infected individuals compared to HIV-uninfected, including cystine, two complex carbohydrates and 3,5-dibromo-l-tyrosine. (PDF 106 kb)

## Abbreviations

ART: antiretroviral therapy
AUC: area under the curve
BAL: bronchoalveolar lavage
HCA: hierarchical cluster analysis
HIV: human immunodeficiency virus
LC-FTMS: liquid chromatography-high-resolution mass spectrometry
*m*/*z*: mass-to-charge ratio
MACS: Multicenter AIDS Cohort Study
MWAS: metabolome-wide association studies
OTU: operational taxonomic units
PCA: principal component analysis
PCoA: principal coordinate analysis
rRNA: ribosomal RNA
sPLS: sparse partial least squares regression
VIP: variable importance on projection
WIHS: Women’s Interagency HIV Study
