# Supplementary material for: Correlation of the lung microbiota with metabolic profiles in bronchoalveolar lavage fluid in HIV infection
Source: Microbiome. 2016 Jan 20;4:3. doi: 10.1186/s40168-016-0147-4 (PMC4721204; doi:10.1186/s40168-016-0147-4)
Supplement: Additional file 2: Figures S1 and S2. — Figure S1. Receiver operating characteristic (ROC) curve using the top discriminatory features between HIV-infected (n = 32) and HIV-uninfected (n = 15) individuals. The top three features (p < 0.0002; FDR 0.05) were used as predictors in a logistic regression model that gave an area under the curve (AUC) = 0.89 and a 10-fold cross-validation classification accuracy of 90.8 % for the 47 training samples and AUC of 0.8 and classification accuracy of 83.3 % for the 12 test set samples. Dotted line training set (n = 47). Solid line validation (test) set (n = 12). Figure S2. Metabolomics of bronchoalveolar (BAL) fluid of HIV-infected individuals compared to HIV-uninfected. Further metabolite analysis demonstrates that four features were significantly over-represented in the bronchoalveolar lavage (BAL) fluid of HIV-infected individuals compared to HIV-uninfected, including cystine, two complex carbohydrates and 3,5-dibromo-l-tyrosine. (PDF 106 kb) [file 40168_2016_147_MOESM2_ESM.pdf]

Additional file 2

Figure Legends:

Figure 1: Receiver operating characteristic (ROC) curve using the top discriminatory features between HIV-infected (n=32) and HIV-uninfected (n=15) individuals. The top 3 features (p<0.0002; FDR 0.05) were used as predictors in a logistic regression model that gave an area under the curve (AUC)=0.89 and a 10-fold cross-validation classification accuracy of 90.8% for the 47 training samples and AUC of 0.8 and classification accuracy of 83.3% for the 12 test set samples. Dotted line: training set (n=47). Solid line: validation (test) set (n=12).

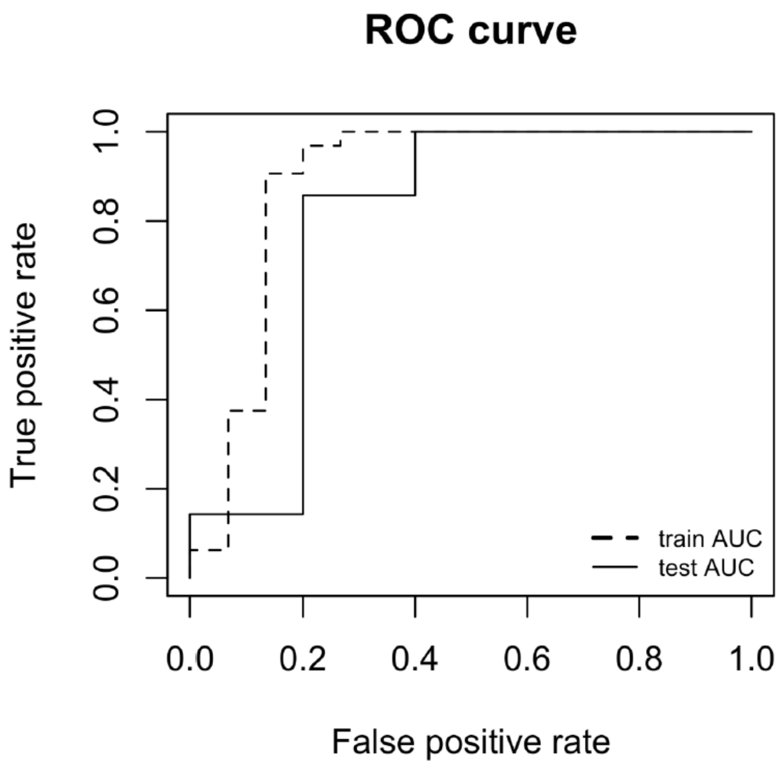

Figure 2: Metabolomics of bronchoalveolar (BAL) fluid of HIV-infected individuals compared to HIV-uninfected. Further metabolite analysis demonstrates that 4 features were significantly over-represented in the bronchoalveolar lavage (BAL) fluid of HIV-infected individuals compared to HIV-uninfected, including cystine, two complex carbohydrates and 3, 5-Dibromo-L-tyrosine.

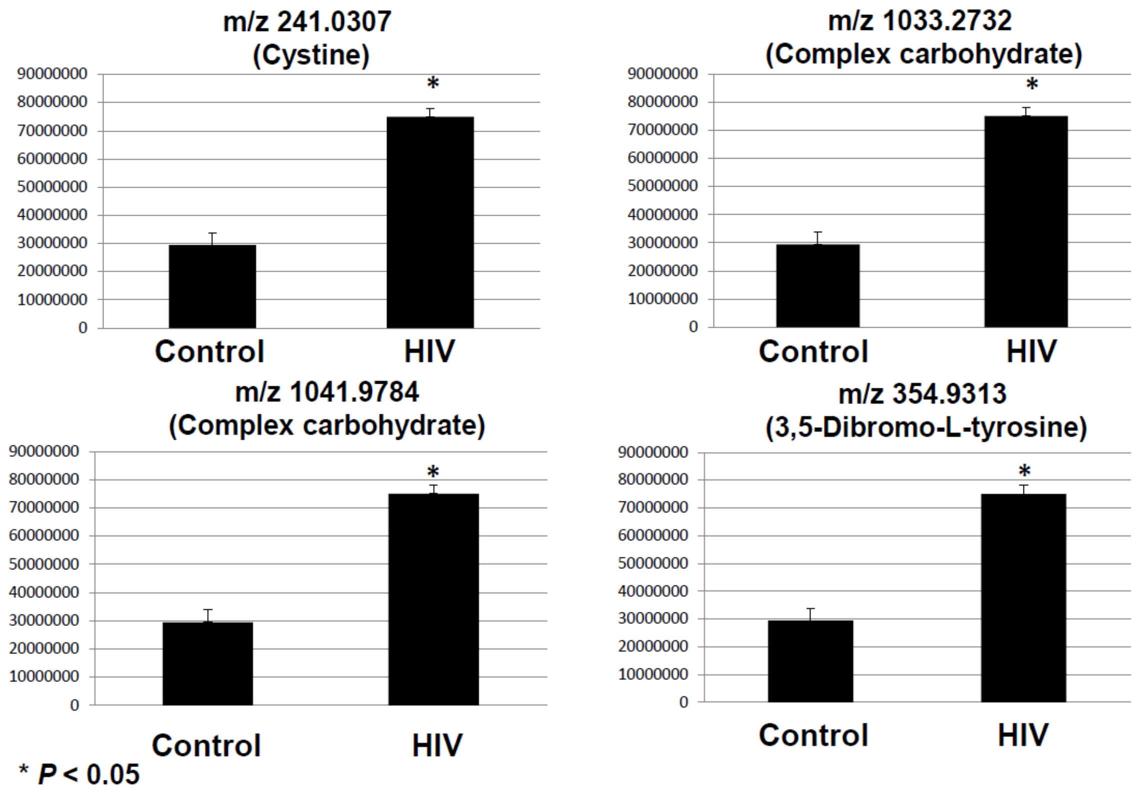

Complete Methods for Metabolomics and Biostatistics and Bioinformatics

Metabolomics

Samples were analyzed by liquid chromatography-high-resolution mass spectrometry (LC-FTMS), as previously described [1]. Briefly, 100 µl aliquots of BAL

fluid were treated with acetonitrile (2:1, v/v) containing an internal standard mix and centrifuged at 14,000 x g for 5 minutes at 4°C to remove protein. These were maintained at 4°C until injection. Mass-to-charge ratio (*m/z*) features were collected by a Thermo Q Exactive hybrid quadrupole-Orbitrap mass spectrometer (Thermo Fisher, San Diego, CA) from *m/z* 85 to 1275 over 10 minutes with each sample analyzed in triplicate. Peak extraction, noise removal, and quantification of ion intensities was performed by an adaptive processing software package (apLCMS) with xMSanalyzer designed for use with LC-FTMS data [2].

Biostatistics and Bioinformatics

The major predictor variable was HIV infection status. Two-sample Wilcoxon rank-sum tests were performed to compare continuous demographic characteristics between HIV-infected and HIV-uninfected individuals. Pearson chi-square tests were used to compare categorical characteristics. Statistical analyses were conducted in NCSS statistical software, except as indicated below. All reported *p*-values were two-sided; and *p*-values of less than 0.05, corrected for multiple hypotheses testing as necessary, were considered statistically significant.

High-resolution mass spectral data were filtered to include only *m/z* features where at least one group (HIV-infected or HIV-uninfected) had values for 50% of the samples. The data were log-transformed and quantile normalized prior to statistical and bioinformatic analyses. Log2 transformation was done to reduce heteroscedasticity and to normalize the data. Quantile normalization of samples was done to minimize sample variability. Batch-effect and sample collection site-effect were corrected using ComBat

[3]. Samples were run in positive ion mode. A total of 5930 *m/z* features were selected after pre-processing and were subsequently used for all statistical analyses 4. LIMMA package [4] in R (Linear Models for Microarray Data) in Bioconductor was used to identify differentially expressed features at a significance threshold of 0.2 after Benjamini-Hochberg false discovery rate (FDR) adjustment. Metabolome data was not adjusted for bronchoalveolar lavage (BAL) dilution; however, concentrations of cystine were adjusted according to BAL and serum urea levels. Two-way hierarchical clustering analysis (HCA) was performed to identify clusters of individuals associated with discriminating clusters of metabolites using the heatmap.2 function in the R package g-plots.

Hierarchical clustering was performed using the built-in hclust ( ) function in R that uses the complete-linkage method for clustering. Weighted UNIFRAC distance was used to cluster the samples by their BAL microbiome while Pearson correlation was used as the dissimilarity measure to cluster samples and their *m/z* features. Principal component analysis using Pirouette version 4.0 (InfoMetrix) was performed, as previously described [5]. Metabolite annotation and pathway enrichment analysis was done using *Mummichog* software implementation of an approach that uses the collective power of combining metabolite identification and metabolic pathway/network analysis into one site. The sparse partial least squares (sPLS) regression method [6] implemented in the R package mixOmics was used to perform integration and visualization of microbiome × metabolome associations. sPLS is a variable selection and dimensionality reduction method that allows integration of heterogeneous omics data from the same set of samples. In this case, metabolome (matrix X) and

microbiome (matrix Z) data were integrated, where X is an $n \times p$ matrix that includes n samples and p metabolites and Z is an $n \times q$ matrix that includes n samples and q bacterial species.

Reference List

1. Johnson JM, Strobel FH, Reed M, Pohl J, Jones DP (2008) A rapid LC-FTMS method for the analysis of cysteine, cystine and cysteine/cystine steady-state redox potential in human plasma. Clin Chim Acta 396: 43-48. S0009-8981(08)00323-9 [pii];10.1016/j.cca.2008.06.020 [doi].

2. Uppal K, Soltow QA, Strobel FH, Pittard WS, Gernert KM, Yu T, Jones DP (2013) xMSanalyzer: automated pipeline for improved feature detection and downstream analysis of large-scale, non-targeted metabolomics data. BMC Bioinformatics 14: 15. 1471-2105-14-15 [pii];10.1186/1471-2105-14-15 [doi].

3. Johnson WE, Li C, Rabinovic A (2007) Adjusting batch effects in microarray expression data using empirical Bayes methods. Biostatistics 8: 118-127. kxj037 [pii];10.1093/biostatistics/kxj037 [doi].

4. Smyth G (2005) Limma: Linear models for microarray data. In: Gentleman VCR, Dudoit S, Irizarry R, Huber W, editors. Bioinformatics and Computational Biology Solutions using R and Bioconductor. 397-420.

5. Soltow Q, Strobel F, Mansfield K, Wachtman L, Park Y et al. (2011) High-performance metabolic profiling with dual chromatography-Fourier-transform mass spectrometry (DC-FTMS) for study of the exposome. Metabolomics. In press.

6. Le Cao KA, Rossouw D, Robert-Granie C, Besse P (2008) A sparse PLS for variable selection when integrating omics data. Stat Appl Genet Mol Biol 7: Article. 10.2202/1544-6115.1390 [doi].
